# 

**Published:** 1833-10

**Authors:** 


### CONTENTS

OF THE

### MEDICAL QUARTERLY REVIEW,

No. I. OCTOBER 1833.

### REVIEWS.

I.

A Treatise on the Diseases of the Eye. By W. LAWRENCE, F.R.S. 1

II.

Ueber den Kropf. Ein Beitrag zur Pathologie und Therapie desselben, von CARL JOSEPH BECK . . . . 17

III.

A Treatise on those Diseases of the Brain and Nervous System which are usually considered and called Mental. By DAVID UWINS, M.D. . 25

IV.

Observations on the Illusions of the Insane, and on the Medico-Legal Question of their Confinement. Translated from the French of M. ESQUIROL, by WILLIAM LIDDELL . . . 39

V.

The Homœopathic Medical Doctrine; or, "Organon of the Healing Art:" a new System of Practice. Translated from the German of S. HAHNEMANN, by CHARLES H. DEVRIENT, Esq.; with Notes, by SAMUEL STRATTEN, M.D. . . . . 49

VI.

A Report of the Method and Results of the Treatment for the Malignant Cholera, by small and frequently repeated Doses of Calomel. With numerous illustrative Cases. By JOSEPH AYRE, M.D. . . 68

VII.

The Principles and Practice of Obstetric Medicine, in a Series of Systematic Dissertations on Midwifery, and on the Diseases of Women and Children. By DAVID D. DAVIS, M.D. . . . 82

VIII.

Outlines of Botany; being a Practical Guide to the Study of Plants. By G. T. BURNETT, Esq. . . . 93

IX.

Observations on Injuries and Diseases of the Rectum. By HERBERT MAYO, F.R.S. . . . . . 105

X.

Nouveau Formulaire Pratique des Hôpitaux. Par MM. MILNE EDWARDS et P. VAVASSEUR, D.D. MM. . . 118

*Reviews continued.*

XI.

Treatise on Diseases of the Skin. By P. Rayer, d.m.p. Translated from the French, by W. B. Dickinson, Esq. . . 121

XII.

A New Exposition of the Functions of the Nerves. By James William Earle, Esq. Part I. . . . . . 124

XIII.

On some Points connected with the Anatomy and Surgery of Inguinal and Femoral Herniæ. By G. T. Guthrie, f.r.s. . . 126

XIV.

A New Dictionary of Medical Science and Literature. By Robley Dunglison, m.d. . . . . . 132

XV.

Graphic Illustrations of Abortion, and the Diseases of Menstruation. By A. B. Granville, m.d. . . . . 135

XVI.

Memoir on the Advantages and Practicability of dividing the Stricture in Strangulated Hernia, on the Outside of the Sac. By C. A. Key, Esq. 139

XVII.

Clinique de l'Hôpital Saint-Louis; ou, Traité complet des Maladies de la Peau. Par M. le Baron J. L. Alibert . . . 144

XVIII.

A Synopsis of Systematic Botany. By Thomas Castle, f.l.s. &c. . 147

XIX.

A Practical Essay on Stricture of the Rectum. By F. Salmon, Esq. . 149

XX.

A Manual of Experiments illustrative of Chemical Science. By John Murray, f.s.a. . . . . . 151

XXI.

Dictionnaire Universel de Matière Médicale, et de Thérapeutique générale, &c. Par F. V. Mérat et A. J. De Lens . . 153

XXII.

Description of an Apparatus intended to facilitate the Treatment of Fractures of the Lower Extremity. By T. M. Greenhow, Esq. . ib.

XXIII.

La Manœuvre de tous les Accouchemens contre Nature, reduite à sa plus grande Simplicité. Par Dr. Jules Hatin . . 154

XXIV.

A Compendious History of Small-pox. By Henry George, Esq. . 155

XXV.

Exposition of the False Medium and Barriers excluding Men of Genius from the P ublic . . . . 156

### Original Communications.

Cases extracted, by permission, from the Note-books of HENRY DAVIES, M.D., Physician-Accoucheur to the Brownlow-street Lying-in Hospital, &c. . . . . . . 157
Observations on Permanent Strictures of the Urethra, by FREDERICK TYRRELL, Esq. . . . . . 160
A Case of Softening of the Brain, with Strumous Tubercles, causing Apoplexy and Palsy. By C. J. ROBERTS, M.D. . . 166
Case of Amputation of the Shoulder-joint, by BRANSBY B. COOPER, Esq. 170
Case of Empyema: with Remarks. Communicated to the Harveian Society, by WILLIAM STROUD, M.D. . . . 174

### COLLECTANEA.

### PATHOLOGY AND PRACTICE.

Cholera cured by Sulphate of Quinine . . . 186
Mistakes as to the Presentation in Labour . . . 188
Inhalation of Chlorine Gas . . . . ib.
Want of Pulse in the Right Arm . . . . 189
Belladonna as a Prophylactic against Scarlet Fever . . ib.
The Radical Cure of Hernia in Turkey . . . ib.
Cholera Morbus on board a French Frigate . . . 190
Remarkable Case of Abortion . . . . ib.
The Hardening System . . . . 191
Case of Traumatic Tetanus . . . . 192
Cases of Easy Parturition in Deformed Women . . ib.

### MISCELLANEOUS.

The Prescriptions in Gölis on Hydrocephalus . . 193
Meat, Drink, and Clothing . . . . 194
Snails . . . . . . ib.
On the Walk of Quadrupeds . . . . 195
The Gunner with the Silver Mask . . . 199
M. Quetelet on the Weight of Man at Different Ages . 204
The Constitution of Pope . . . . 205
On Dissecting and Preparing Animals for Collections. By Professor CARUS of Dresden . . . . . 207
Commercial Travellers . . . . 208
Geological Distribution of the Musci . . . 209
Habits of Mercantile Men . . . . 210
On the simultaneous Presence of Prussian Blue, and of a Saccharine Matter, in a particular Variety of Human Urine. By M. CANTU . 211

*Collectanea continued.*

Experiments on Hemlock and Henbane . . . 215
Marchantia Polymorphia . . . . 216
Arrowroot . . . . . ib.
The Itch . . . . . . . 218
Longevity of Authors . . . . 219

### Medical Politics and Intelligence.

The College of Physicians . . . . 221
Royal College of Surgeons in London . . . 224
The Aldersgate-street Dispensary . , · 226

### OBITUARY.

Dr. Darwall. Dr. Gordon Smith . . . 228

Meteorological Register. Notices . . . ib.

